# Plant origin and ploidy influence gene expression and life cycle characteristics in an invasive weed

**DOI:** 10.1186/1471-2229-9-33

**Published:** 2009-03-23

**Authors:** Amanda K Broz, Daniel K Manter, Gillianne Bowman, Heinz Müller-Schärer, Jorge M Vivanco

**Affiliations:** 1 Center for Rhizosphere Biology, Colorado State University, Fort Collins, CO 80523-1173, USA; 2 Department of Horticulture and Landscape Architecture, Colorado State University, Fort Collins, CO 80523-1173, USA; 3 USDA-ARS, Soil-Plant-Nutrient Research Unit, Fort Collins, CO 80526, USA; 4 Département de Biologie/Ecologie & Evolution, Université de Fribourg/Pérolles, Chemin du Musée 10, CH-1700 Fribourg, Switzerland

## Abstract

**Background:**

Ecological, evolutionary and physiological studies have thus far provided an incomplete picture of why some plants become invasive; therefore we used genomic resources to complement and advance this field. In order to gain insight into the invasive mechanism of *Centaurea stoebe *we compared plants of three geo-cytotypes, native Eurasian diploids, native Eurasian tetraploids and introduced North American tetraploids, grown in a common greenhouse environment. We monitored plant performance characteristics and life cycle habits and characterized the expression of genes related to constitutive defense and genome stability using quantitative PCR.

**Results:**

Plant origin and ploidy were found to have a significant effect on both life cycle characteristics and gene expression, highlighting the importance of comparing appropriate taxonomic groups in studies of native and introduced plant species. We found that introduced populations of *C. stoebe *exhibit reduced expression of transcripts related to constitutive defense relative to their native tetraploid counterparts, as might be expected based on ideas of enemy release and rapid evolution. Measurements of several vegetative traits were similar for all geo-cytotypes; however, fecundity of tetraploids was significantly greater than diploids, due in part to their polycarpic nature. A simulation of seed production over time predicts that introduced tetraploids have the highest fecundity of the three geo-cytotypes.

**Conclusion:**

Our results suggest that characterizing gene expression in an invasive species using populations from both its native and introduced range can provide insight into the biology of plant invasion that can complement traditional measurements of plant performance. In addition, these results highlight the importance of using appropriate taxonomic units in ecological genomics investigations.

## Background

Plant invasion into new environments is an extremely costly problem, not only monetarily but also ecologically. Invasive plant infestations reduce biodiversity by displacing native species and can literally destroy some native ecosystems by altering important ecosystem characteristics [1]. However, the reasons why some plants remain at low abundance in their home range but become dominant in their new range is not well understood and remains one of the most perplexing questions in ecology. Multiple non-exclusive hypotheses have been proposed to explain plant invasion into new environments [2].

A long standing idea in the field of invasion biology is that of enemy release [3]. This hypothesis posits that introduced plants escape their native co-evolved specialist enemies, which allows them to rapidly increase their numbers [3]. Blossey and Notzold (1995) proposed the evolution of increased competitive ability (EICA) hypothesis, which builds on the idea of enemy release and has generated much interest in recent years [4]. The EICA hypothesis suggests that costly defense against specialists no longer enhances fitness of plants in the introduced range; therefore introduced plants will evolve to put fewer resources into defense allowing them to increase allocation of resources towards growth and reproduction [4]. This hypothesis has been supported by experimental evidence, but only in part [5]. Multiple refinements to the EICA hypothesis have been proposed to account for altered selective pressures in the new environment including the presence of generalist enemies [6-9] and changes in resource availability [10,11].

The majority of studies examining EICA and other hypotheses of plant invasion have focused on ecological, physiological and to some extent chemical plant characteristics [2,5,12,13]. However, with the current revolution in genomics technologies, the question arises as to whether ecological phenomena such as plant invasion can be better understood by studies of genetics or gene expression profiling. The development of genomics resources for non-model species of invasive weeds is increasingly becoming possible as new technologies become more available and affordable, as demonstrated by Broz et al. 2007 (spotted knapweed) and Anderson et al. 2007 (leafy spurge), aiding in the ability of researchers to investigate the biology of invasive weeds [14,15]. In regards to ecological hypotheses, it may be particularly useful to characterize expression of genes related to plant defense and competitive ability.

Recently, an EST (expressed sequence tag) library resource was developed for the problematic invasive plant, *Centaurea stoebe *L. (Gugler) Hayek (also known as *C. maculosa *Lam, *C. biebersteinii*, spotted knapweed) [15]. *C. stoebe*, a native to Eurasia, is able to invade not only ruderal habitats, but also rangelands, pastures and prairies in North America, where it often establishes dense monocultures and excludes native plant species. *C. stoebe *first appeared on both coasts of North America around the late 1800s [16,17], and has since greatly expanded its range to all but three states in the continental US [18].

Molecular marker studies revealed relatively large amounts of genetic diversity within and among populations in both the native and introduced ranges [19,20], and suggest that this species has been introduced to North America multiple times. Thus, genetic drift resulting from bottle-necks or founder effects does not seem to have played an important role in the invasive success of this weed. Extensive field collections thus far conclude that the native range consists of morphologically indistinguishable diploid (2n = 2x = 18; *C. stoebe *ssp *stoebe*) and tetraploid (2n = 4x = 36; *C. stoebe *ssp *micranthos*) forms of the weed [21] that occasionally occur in mixed stands [22]. In the introduced range, populations had been found to contain the tetraploid form exclusively [21] until a recent extensive survey identified a single mixed stand of diploid and tetraploid plants in western Canada [22]. This suggests that both forms of the weed were introduced, but only the tetraploid has become an invasive problem [22].

*C. stoebe *is able to tolerate a wide variety of soil types and precipitation amounts in both Eurasia and North America [21,23]. Robust cross-continental comparisons have provided empirical evidence for a niche shift between native and introduced populations [24], and more recently between native and introduced tetraploid *C. stoebe*, with the invasive tetraploids occurring in drier and warmer climates [22]. Moreover, the range of the native tetraploid in Eurasia has expanded over the range of the native diploid within the past 100–150 years [21], and introduced tetraploids appear to have a higher ecological tolerance, or niche breadth, than either of the native forms [22,24]. Thus, the invasive success of *C. stoebe *appears to be partially due to pre-adaptation of the native tetraploid cytotype to drier climates, a trait which has been further selected for in the introduced range [22]. However, more studies are needed to rule out other alternatives related to the weeds invasive success.

Both diploid and tetraploid forms of *C. stoebe *are out-crossing, insect-pollinated asters, but the diploid tends to have a biennial monocarpic life cycle, whereas the tetraploid tends to be a polycarpic perennial, continuing to flower over multiple growing seasons [21,22,25]. Compared to native populations, introduced tetraploids exhibit the highest proportion of polycarpic plants and have the greatest number of stems per plant [22], which may increase their reproductive capacity. It is hypothesized that this perennial polycarpic life cycle is selected for, particularly in environments lacking natural enemies [9], which may help explain why the tetraploid form became predominate in the introduced range.

Although there are a small number of studies that examine ploidy differences between native and introduced populations of plants, this factor is most often unaccounted for in ecological studies of invasive weeds [5], including *C. stoebe*. Many of the worst weeds are polyploids, and changes in plant ploidy may lead to changes in life history traits, genetic diversity, gene expression or capacity for adaptation and evolution [26]. Therefore, in a comparison of plants from both the native and introduced range, it is important to compare the same taxonomic unit [5], and understand differences between taxonomic units.

As it appears that both ploidy pre-adaptation (European diploid vs. tetraploid) and selection (European vs. North American tetraploid) may be important factors in *C. stoebe *invasion, we were interested in characterizing the three distinct geo-cytotypes of *C. stoebe*: native diploids, native tetraploids and introduced tetraploids. We grew plants from multiple populations, representing each of the three geo-cytotypes in a common environment and monitored plant performance characteristics and life cycle habits. In addition, we identified gene sequences in the *C. stoebe *EST library that may be involved in constitutive basal plant defense or rapid evolution, as these traits may be important in the plants invasive success. Expression of these genes was characterized in each geo-cytotype using quantitative PCR.

Based on ideas of enemy release and rapid evolution of plants in the introduced range, and on trends in polyploidy, we developed hypotheses concerning plant performance and gene expression of the geo-cytotypes. First, we hypothesized that introduced tetraploids would exhibit reduced expression of constitutive defense and secondary metabolite related genes, but an increase in plant performance when compared to native tetraploids, due to a partial release from enemies. Second, we also expected that genes involved in genome stability would be expressed to a greater extent in introduced versus native tetraploids due to possible novel environmental stresses experienced in the introduced range. Although evolution is predominately thought to be due to random mutations, there is some evidence that expression of transposable elements and DNA repair enzymes influence genetic stability and stress-induced evolutionary strategies in organisms [27-29]. Therefore, we also assessed transcript accumulation of two active transposable elements and a DNA repair enzyme, which might facilitate rapid evolution in a new environment. Finally, we hypothesized that native tetraploids would exhibit increased expression of genes involved in secondary metabolite production compared to diploids, due to potential increases in the metabolic activities of polyploids [30].

## Results

### Plant performance and life cycle analysis

**Figure 1:**
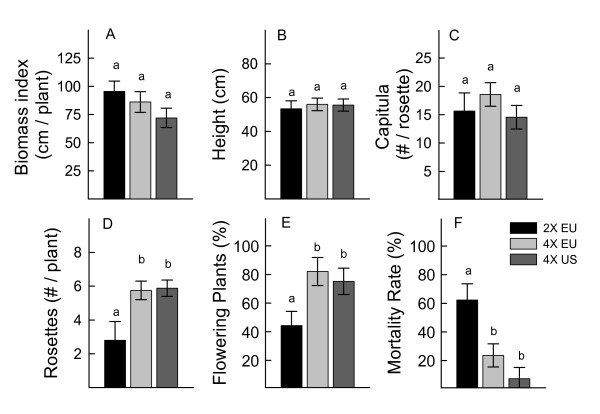
**Plant performance and life-cycle traits of *C. stoebe *geo-cytotypes**. *C. stoebe *plants were grown from seed in a common greenhouse environment. Plants were measured for leaf length and leaf number while in rosette form, and these values were multiplied to obtain an early indicator of biomass (A). After bolting, stem height (B) of each bolting plant was measured the day the first flower opened and the number of capitula per flowering plant (C) were counted after the stems had senesced. The number of newly formed rosettes after flowering (D), the percent of flowering individuals (E), and the percent mortality after flowering (F) were monitored. Legend; 2× EU, native Eurasian diploid populations; 4× EU, native Eurasian tetraploid populations; 4× US, invasive North American tetraploids. Significant differences in plant traits were determined for geo-cytotypes of interest (EU 2× versus EU 4× and EU 4× versus US 4×) using pair-wise comparisons of LSmeans. Bars represent LSmeans and standard errors. Fisher's LSD was used for pair-wise mean comparisons. Different letters above the columns indicate significant differences (P < 0.05) between pairs of geo-cytotypes.

**Figure 2:**
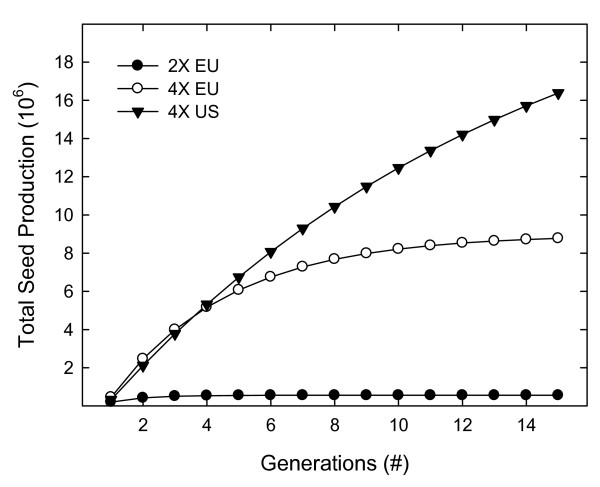
**Simulation of total seed production over time**. The simulation followed a cohort of 1000 plants over time assuming that the number of flowering plants for each generation was 75.2, 82.1, and 44.3% (4× US, 4× EU, and 2× EU, respectively) of the total population (Figure 1E); and each generation the number of flowering plants declined according to a mortality rate of 7.3, 23.6, and 62.3% (4× US, 4× EU, and 2× EU, respectively) as shown in Figure 1F. For each flowering plant, the total number of seeds was estimated as the product of the number of new rosettes per plant (5.88, 5.75, and 2.8 for the 4× US, 4× EU, and 2× EU, respectively; Figure 1D), number of capitula per rosette (14.6, 18.6, and 15.7 for the 4× US, 4× EU, and 2× EU, respectively; Figure 1C), and 30 seeds per capitula [17]. Legend; 2× EU, native Eurasian diploid populations; 4× EU, native Eurasian tetraploid populations; 4× US, invasive North American tetraploids. Refer to Additional file 1: Table 1 for the mean values used in this analysis.

No significant differences in vegetative plant performance characteristics were found between *C. stoebe *geo-cytotypes (Figure 1, Additional File 1: Table 1). Before bolting, the plant biomass index tended to be higher in diploid populations than in tetraploids, but the results were not significant (Figure 1A). Similarly, stem height was not different between the three geo-cytotypes (Figure 1B). However, differences in life cycle were noted between ploidy groups; a higher percentage of both native and invasive tetraploid plants flowered in the first year compared to the diploid plants (Figure 1E). Fewer than half of the diploid plants flowered in their first year of growth, and over 60% died after flowering (Figure 1F, Additional File 1: Table 1). In comparison, over 75% of both native and introduced tetraploids flowered their first year and only 24% and 7% died after flowering, respectively (Figure 1E, F, Additional File 1: Table 1). In addition, tetraploids produced more new rosettes after senescence of the parent plant than diploids (Figure 1D). Interestingly, the number of capitula per plant (Figure 1C) was not different between the three geo-cytotypes. The observed differences in life cycle characteristics reflect the moncarpic life cycle of the diploid and the polycarpic life cycle of the tetraploid [21], and are likely important in plant population fecundity over time, as illustrated by a simulation of seed production (Figure 2). Over a fifteen-year period, this simluation estimates production of 0.6, 8.8, and 16.4 million seeds for populations of the native diploid, native tetraploid, and introduced tetraploid, respectively (Figure 2).

### Gene expression analysis

**Figure 3:**
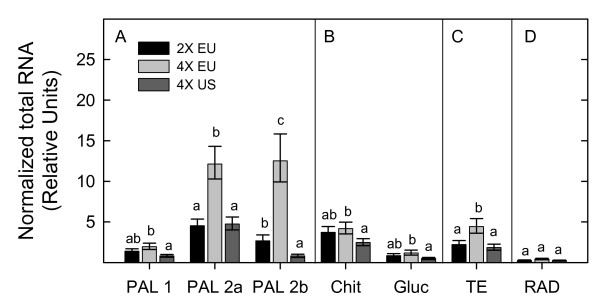
**Gene expression profiles of *C. stoebe *geo-cytotypes**. For each sample, total RNA (ng/ul) was estimated using the appropriate standard curve for each gene of interest and normalized using the geometric mean of the standards actin, cytochrome c oxidase, and ubiquitin as suggested in Vandersompele et al. 2002 [61]. Significant differences in gene expression (log cDNA) were determined for geo-cytotypes of interest (EU 2× versus EU 4× and EU 4× versus US 4×) using pair-wise comparisons of LSmeans. Bars represent back-transformed LSmeans and standard errors. Fisher's LSD was used for pair-wise mean comparisons, and values are reported in Table 1. Different letters above the columns indicate significant differences (P < 0.05) between pairs of geo-cytotypes. Legend; 2× EU, native Eurasian diploid populations; 4× EU, native Eurasian tetraploid populations; 4× US, invasive North American tetraploids. Panel A: Genes involved in secondary metabolism; PAL (Phenylalanine ammonia lyase) 1, 2a, 2b. Panel B: Genes involved in defense response; Chit (chitinase) and Gluc (glucanase); Panel C: Gene involved in transposition; TE (transposable element); Panel D: Gene involved in DNA repair and recombination, RAD.

Tetraploid plants from the introduced range had significantly lower rates of gene expression for all three PAL transcripts compared to tetraploid plants from the native range, providing evidence in favor of our hypothesis (Figure 3A). PAL1 transcript accumulation in introduced tetraploids was 2.4 times lower than the amount in native tetraploids, whereas PAL2a and PAL2b were 2.6 and 16.7 times lower, respectively (Table 1). PAL 1 expression was lower than expression for either form of PAL 2 in all geo-cytotypes (Figure 3A). Similarly, glucanase transcripts showed over a two-fold reduction in expression in introduced tetraploids than their native counterparts (Figure 3B, Table 1). Chitinase expression was 1.7 fold lower in introduced tetraploids than native tetraploids (Table 1). In general, expression of all tested secondary metabolism- and defense-related transcripts was lower in tetraploids from the introduced range compared to their native counterparts.

**Table 1:** Relative gene expression values of *C. stoebe *geo-cytotypes.

	**EU 2× vs EU 4×**		**Relative Expression**			**EU 4× vs US 4×**	
**Gene**	**t**	**p-value**	**EU 2×**	**EU 4×**	**US 4×**	**t**	**p-value**

Actin	0.84	0.411	0.80^a^	1.00^a^	0.69^a^	1.41	0.174
COX	0.96	0.348	1.25^a^	1.00^a^	0.86^a^	0.63	0.538
UBQ	0.84	0.413	1.24^a^	1.00^a^	1.07^a^	0.26	0.795
PAL 1	1.20	0.245	0.71^ab^	1.00^b^	**0.42**^a^	**3.06**	**0.006**
PAL 2a	**4.91**	**<0.001**	**0.37**^a^	1.00^b^	**0.39**^a^	**4.00**	**<0.001**
PAL 2b	**8.19**	**<0.001**	**0.21**^b^	1.00^c^	**0.06**^a^	**8.19**	**<0.001**
Chitinase	0.47	0.644	0.89^ab^	1.00^b^	**0.59**^a^	**2.14**	**0.045**
Glucanase	0.90	0.373	0.72^ab^	1.00^b^	**0.41**^a^	**2.42**	**0.025**
TE	**2.41**	**0.025**	**0.50**^a^	1.00^b^	**0.42**^a^	**3.06**	**0.006**
RAD	1.55	0.136	0.61^a^	1.00^a^	0.57^a^	1.78	0.090

For each sample, total RNA (ng/ul) was estimated using the appropriate standard curve for each gene of interest and normalized using the geometric mean of the three standards: actin, cytochrome c oxidase (COX) and ubiquitin (UBQ), as suggested in Vandersompele et al. 2002 [61]. Genes of interest included three isoforms of PAL (phenylalanine ammonia lyase) 1, 2a, 2b, involved in secondary metabolism; chitinase and glucanase, involved in defense response; and a transposable element (TE) and DNA repair/recombination gene (RAD), potentially involved in rapid evolution. Geo-cytotypes are 2× EU, native Eurasian diploid populations; 4× EU, native Eurasian tetraploid populations; 4× US, invasive North American tetraploids. Significant differences in gene expression (log cDNA) were determined for geo-cytotypes of interest (EU 2× versus EU 4× and EU 4× versus US 4×) using pair-wise comparisons of LSmeans. LSmeans were back-transformed and expression values are shown relative to native Eurasian tetraploid populations (4× EU). Fisher's LSD and absolute t values are reported for each pair-wise comparison.

Contrary to our second hypothesis, introduced tetraploids showed over two-fold less expression of a transposable element (CACTA En/Spm subclass) transcript than native tetraploids (Figure 3C). The other transposable element (mutator subclass) showed extremely low levels of transcript accumulation in most samples, nearly all of which fell below the standard curve for that gene (data not shown). Of the usable values, the data suggested that introduced populations expressed this transposable element to a lower extent than native populations, but the sample size was very low and thus overall values may not accurately reflect expression in these populations. Expression of RAD was low in all plant types, but also showed the highest relative mean expression in native tetraploids, although this result was not significant (Figure 3D, Table 1).

Diploid and tetraploid plants from the native range showed similar relative expression levels for seven out of ten genes tested; PAL1, glucanase, chitinase, RAD, and the three housekeeping genes (Figure 3A, B, D, see Additional File 2: Figure 1 for housekeeping gene profiles, Table 1). Expression of PAL2a and PAL2b was higher in native tetraploids compared to diploids (Figure 3A, Table 1) as hypothesized. Expression of CACTA transposable element was also higher in native tetraploids compared to diploids (Figure 3C, Table 1). Introduced tetraploids showed similar expression profiles when compared to diploids for nine of the ten genes tested (Figure 3). The expression of PAL2b was over three fold lower in introduced tetraploids compared to diploids (Table 1).

## Discussion

### Plant performance and life cycle analysis

Ridenour et al. (2008) recently reported that in a common garden in Montana, *C. stoebe *plants from North America exhibit greater biomass, tougher leaves and increased trichome density when compared to their Eurasian counterparts [31]. Based on this finding and hypotheses such as EICA that suggest invasive plants may evolve to increase resource allocation to growth [4], we expected that introduced tetraploids would out-perform both native diploids and tetraploids. However, in our study, neither of the plant vegetative growth characteristics examined (biomass index and stem height, Figure 1A, B), showed a significant difference. Ridenour et al. (2008) performed the bulk of their experiments on populations with unknown ploidy; however, one experiment containing plants of known ploidy revealed greater rosette diameters of introduced tetraploids compared to native tetraploids [31]. Conversely, Müller et al. (1989) observed that Hungarian and German diploids had greater dry weights and shoot diameters than North American tetraploids when grown in a European soil, but sample sizes were relatively small [25]. The observed differences may be due to the various populations chosen, the type and origin of soil used (ie; North American soil [31] versus European soil [22,25] present study), or other factors involved in each of the above studies. These inconsistencies may suggest that vegetative growth is not the best indicator of invasiveness.

As previously noted by Müller (1989), life cycle differences between *C. stoebe *geo-cytotypes may have greater relevance to fitness than single performance traits [25]. In the first year of this study, flowering plants of all geo-cytotypes had a similar number of capitula (Figure 1C): however, fewer diploid plants flowered in the first year of growth than tetraploids, diploids formed fewer new rosettes, and diploids suffered greater mortalities after flowering (Figure 1D, E, F). In combination these measures suggest that the reproductive capacity of tetraploids is greater than that of diploids. Additionally, we expect introduced tetraploid populations to have a higher reproductive capacity when compared to the native tetraploids, as illustrated by a simulation of seed production (Figure 2). Ongoing experiments will provide more complete information about the life-cycle of these plants and seed production over their entire life span. Thus, although we did not detect any significant differences in vegetative traits between *C. stoebe *geo-cytotypes, there is some indication of a long-term difference in plant fecundity, with the invasive tetraploid showing highest performance of the three geo-cytotypes studied.

### Gene expression analysis

#### Secondary metabolism and defense

We selected three distinct PAL unigenes for analysis of secondary metabolite-related transcript, as this enzyme represents the first enzymatic step in the flavonoid synthesis pathway which contributes isoflavones, anthocyanins, condensed tannins and other secondary metabolic compounds in plants [32-34]. Flavonoids are often stored in plant tissues as 'pre-formed' defense compounds and may act as pathogen and herbivore deterrents [33]. The expression of PAL gene transcripts in addition to the secondary metabolites resulting from the flavonoid pathway are known to be important in plant defense against pathogens, herbivores and environmental stresses [32-34].

A chitinase and a beta-1,3-glucanase were selected to analyze defense-related transcription, as these transcripts represent members of the PR family of proteins, which have been widely implicated in plant resistance to pathogens [35-37]. Different forms of chitinase are involved in both active and passive defense responses in plants [37]. Glucanases have also been implicated in plant resistance to pathogens, and beta-1, 3-glucanases comprise part of the PR-2 group of pathogenesis-related genes [35].

The fact that PAL, chitinase and glucanase transcripts were all reduced in introduced tetraploids compared to native tetraploids (Figure 3A,B) might suggest that populations of plants from the introduced range will be less defended against herbivores than natives, as is generally predicted by the EICA hypothesis. Some studies suggest that constitutive or basal levels of defense-related transcripts in plants, similar to those analyzed in this study, can be used to predict pathogen susceptibility and induced defense responses [38,39]. Very subtle genetic mutations, such as those in the *Arabidopsis *cpr (constitutive expressers of pathogenesis related genes) mutant, have been shown to increase basal levels of systemic acquired resistance, which in turn increase levels of pathogen resistance [38]. In addition, the over-expression of PR proteins *in planta *typically results in a phenotype of enhanced disease resistance [38,40,41]. Plants with high constitutive defenses may, however, also have a lower degree of defense induction than those with low constitutive defenses [10,12].

Recent reports indicate that introduced *C. stoebe *plants are better defended against both generalist and specialist enemies than natives [31]. This observation, in combination with the current study, may suggest that introduced populations have a higher potential degree of defense induction. However, the current study only measured levels of genes that may be involved in constitutive defense. Thus, our results must be interpreted with caution with regard to ecological hypotheses of plant defense in biological invasions.

It is important to note here that the release of *C. stoebe *from specialist enemies has been considered an important factor in the invasive success of the weed, and this has spurred the introduction of a number of biological control species to North America over the past thirty years [9,16,42,43]. Although many of these specialist herbivores have become established and widespread, *C. stoebe *densities have only been reduced in a few specific areas (e.g[44]), and the weed continues to expand its range at other sites [9,23]. Interestingly, field observations in North America suggest that introduced *C. stoebe *experiences little pressure from generalist herbivores and pathogens (RM Callaway and WM Ridenour, personal communication), indicating that *C. stoebe *currently experiences a partial release from both specialist and generalist enemies in the introduced range.

In order to better understand defense responses in *C. stoebe*, future studies should monitor gene expression and physiological responses in tetraploid geo-cytotpyes when exposed to pathogens and herbivores. This would help determine if expression of genes involved in constitutive defenses are good predictors of pathogen and herbivore susceptibility in *C. stoebe*. In addition, it would be interesting to test the response of *C. stoebe *geo-cytotypes to a variety of generalist and specialist enemies at the level of gene expression.

#### Evolutionary capacity

The activity of transposable elements could facilitate evolution by reorganizing the genome, and may be one important aspect in this process [27,28]. Therefore, we hypothesized that introduced populations of *C. stoebe *would have the highest expression of the transposable elements analyzed, potentially due to novel stresses encountered in the introduced range. However, this was not the case. In fact, native tetraploid populations had the highest expression rate of one CATCA En/Spm subclass transposable element (Figure 3C). The expression of RAD, which is involved in DNA recombination/repair [45], was also highest in native tetraploid populations, but was not significantly different from that of introduced populations (Figure 3D).

Although the expression of transposable elements could facilitate rapid evolution, transposition may not be adaptive and could cause deleterious genomic rearrangements as opposed to beneficial ones. In other studies, certain transposable elements have been detected in plants at specific growth stages or under conditions of biotic and abiotic stress [46,47]; however, the biological role of active transposition currently remains unclear. Additionally, recent evidence suggests that epigenetic mechanisms such as DNA methylation and chromatin remodeling can play an important role in the regulation of gene expression in polyploids which may facilitate adaptive plasticity [48-50]. Similarly, paramutation (interactions between homeologous genetic loci) can also result in differential regulation of genes between polyploids and their diploid progenitors [48,50]. Thus, although we did not detect the changes we predicted in expression of transposable elements, it is entirely possible that factors other than chromosomal rearrangement through transposition are responsible for the observed changes in gene expression.

#### Plant ploidy

Although plant ploidy is often unaccounted for in comparisons of native and introduced populations, we found it to be a necessary and essential component for gene expression analyses. In native populations, we found lower expression of PAL2a, PAL2b and the transposable element in diploids compared to tetraploids, and all other genes examined showed similar relative expression (Figure 3, Table 1). The literature suggests that gene expression rates in polyploids tend to vary depending on plant species, ploidy, genetic background, and the genes examined; however, the phenomenon of gene dosage compensation appears to be common [49,51-53]. This dosage effect results in gene or protein expression patterns in polyploids which are similar to their diploid progenitors. We did not necessarily expect to see this phenomenon in our plant populations because other studies involving ploidy and gene or protein expression have traditionally utilized plants with the same genetic background [49,51,52], whereas evidence suggests that *C. stoebe *plants within the native range harbor different genetic backgrounds [19,20]. However, it appears that gene dosage compensation may be occurring to some extent in the native cytotypes of *C. stoebe*. Additionally, we observed increased expression of two PAL transcripts in native tetraploids compared to diploids, which may reflect increases in secondary compounds due to polyploidy as is seen in other plants [30].

Interestingly, native diploids exhibited similar expression profiles for nine of the ten total genes analyzed when compared to introduced tetraploids (Figure 3, Table 1), also suggesting gene dosage compensation. This result was rather surprising in that the diploid appears to be extremely rare (i.e., unsuccessful) in the introduced range, whereas the introduced tetraploid is a very problematic weed. Therefore, it is likely that other factors, such as plant performance characteristics, life cycle traits and the expression of other genes, are of greater importance in determining the success of tetraploids over diploids in the introduced range. Overall, the observed differences in gene expression between and within ploidies highlights the importance of using appropriate plant types when examining a particular species in both the native and introduced range.

#### Alternative gene roles and regulation

Genes similar to those selected in the current study have been detected in response to a variety of cues and conditions that do not necessarily reflect their primary annotation. For instance, many genes involved in defense response [54], flavonoid biosynthesis [34] and active transposition [46,47] have been detected during particular points of plant growth and development. In this study we attempted to minimize any possible developmental differences in gene expression by sampling expanded, fully developed rosette leaves of similar age from all plants. All of the plants were grown in the same greenhouse environment and at the time of sampling remained in rosette form, none showing signs of bolting. If the genes tested here were expressed predominantly in response to developmental cues, it could be expected that expression of transcripts would be extremely similar across all geo-cytotypes, which is not what was observed.

Additionally, it is possible that the defense genes analyzed in this study are important for aspects other than plant defense against enemies. For instance, the production of certain flavonoids are thought to play important roles in photo-protection, frost hardiness and drought resistance [33], which could influence expression of PAL genes. *C. stoebe *occupies areas in both the native and introduced range that are often subject to these types of abiotic stress [21,22,24]. Thus, expression of PAL transcripts and resulting flavonoid accumulation may be important in both the biotic and abiotic stress response of the plant.

## Conclusion

Although we sampled only a small subset of genes, we identified differences in gene expression between native and introduced populations of plants that may have ecological relevance. We found that introduced tetraploids exhibited lower expression of constitutive defense genes than native tetraploids, as might be predicted based on general ideas of enemy release and rapid evolution. Plant origin and ploidy were found to have a significant effect on both life-cycle characteristics and gene expression. This highlights the importance of determining plant ploidy in ecological and genomics investigations, and suggests that *C. stoebe *invasion can be influenced by both plant ploidy and altered gene expression in the introduced range.

We have demonstrated that the quantitative analyses of gene expression in native and introduced plant populations reveal trends that may provide additional insight into ecological hypotheses. However, the mechanisms underlying the observed changes in gene expression remain unclear, and further work is needed in this area. A better understanding of the genetic and molecular basis of invasiveness in exotic plants is not only an interesting case study in evolution, but is important to further our understanding how these invasions occur, and to choose appropriate management interventions. The techniques used in our study can provide an important complement to classical ecological measurements of plant fitness and competitive success.

## Methods

### Centaurea field sampling, greenhouse experiment and tissue sample collection

#### Field Sampling

Populations of *C. stoebe *were sampled in Eurasia and North America during summer and fall of 2005 using a transect method ([22] Table 2). One fifty-meter-long transect was chosen as the basic sampling unit for each population. Sixteen plants were sampled systematically every three meters (starting at 2.5 m and ending at 47.5 m) along each transect. At each sampling point, seeds were taken from the nearest fruiting plant. For each population, GPS coordinates were recorded. Seeds from each maternal plant were labeled and kept separate. Ploidy was determined for each population by growing four to sixteen seedlings from different parents and analyzing the nuclear DNA content using flow cytometry [22]. Although other populations were collected as part of this larger experiment, only populations that were sampled using the transect method and only those found to have exclusively diploid or tetraploid individuals (not mixed stands) were used in subsequent gene expression analyses. In total, plants of seven diploid and eight tetraploid populations from Eurasia, and of eight tetraploid populations from North America were utilized; these are referred to as geo-cytotypes (populations listed in Table 2).

**Table 2:** Plant origin and ploidy of studied *C. stoebe *populations

Continent NA: North America EU: Eurasia	Ploidy	Country or State	Pop	Locality	Longitude	Latitude
NA	4×	Montana	MT 1	Missoula	-114.1008929	46.82048877
NA	4×	Montana	MT 2	Florence, Bitteroot Reserve	-114.1406713	46.58378483
NA	4×	Montana	MT 3	Ross Hole	-113.9748996	45.83464729
NA	4×	Montana	MT 10	Missoula, Blanchard Flat	-113.3832243	46.99937593
NA	4×	Montana	MT 11	Dixon, Moeise	-114.2997544	47.30836457
NA	4×	Oregon	OR 1	Portland, Rivergate	-122.7701958	45.61806134
NA	4×	Oregon	OR 3	Dee Flat	-121.6293944	45.5897611
NA	4×	Oregon	OR 11	Cougar Reservoir	-122.26225	44.15666
EU	4×	Hungary	H 2	Devecser, Zergeboglaros	17.44339689	47.11656667
EU	4×	Hungary	H 4	Barcs	17.49997063	45.96521169
EU	4×	Ukraine	UA 4	Khotyn	26.46580403	48.51591216
EU	4×	France	FRA 2	St-Clément-de-rivière	3.858896331	43.71806565
EU	4×	Germany	DE 3	Nürnberg	11.08564915	49.41683985
EU	4×	Germany	DE 4	Steinbach, Baggersee	10.63143809	49.99367438
EU	4×	Switzerland	CH 1	Grontenswill-Zetwill	8.15126773	47.28327703
EU	2×	Austria	AT 3	Hainburg	16.95549745	48.15341312
EU	2×	Switzerland	CH 4	Ausserberg	7.84454	46.31189
EU	2×	Germany	DE 1	Simbach am Inn	13.01505128	48.26064449
EU	2×	France	FRA D	St-Cirq Lapopie	1.679543126	44.46250283
EU	2×	Hungary	H 3	Tapolca	17.33497261	46.91410163
EU	2×	Hungary	H 6	Kiskunfelegyhaza	19.89586137	46.70589072
EU	2×	Ukraine	UA 2	Olesko	24.83581002	49.93014257

#### Greenhouse experiment

In May 2006, five seeds from each maternal plant were placed in multi-pot trays in a mixture of sand (20%) and compost (80%, made from yard waste at the Botanical Garden in Fribourg, Switzerland). The greenhouse was not heated but temperatures stayed above 0°C in winter. One plantlet per mother plant was re-potted at eight weeks in 1 L pots of sandy soils (20% sand, 80% compost) in a naturally lit greenhouse supplemented with artificial light. The greenhouse was located near the University of Fribourg, Switzerland. Plants were watered regularly, but were not given nutrient solution. Number of leaves and longest leaf length were measured three times (10^th^–14^th ^July 2006, 7^th^–11^th ^August 2006, 27^th ^April–3^rd ^May 2007) before plants started bolting. Number of leaves multiplied by the longest leaf size was used as a non-destructive proxy for plant biomass, and is referred to subsequently as "biomass index". When the first flower opened (6^th ^July–23^rd ^August 2007), the date, number of stems, height of stems and number of buds larger than 5 mm were recorded for each plant. Survival, number of capitula per flowering plant and number of newly formed rosettes were estimated once the stem had senesced at the beginning of October 2007. The percent of flowering plants and percent plant mortality was calculated for each population. Previous studies on *C. stoebe *have indicated that although environmental maternal effects on offspring are detectable, they are relatively weak compared to other factors such as plant genotype and environmental conditions [55], therefore we do not expect maternal effects to confound the experimental results.

#### Tissue sampling

In November 2006 all plants remained in rosette form and had not bolted. One fully developed undamaged leaf was removed from each chosen plant using a razor blade. A few plants had minimal herbivore damage on the leaves, and these plants were avoided during tissue sampling. Four plants were sampled from each chosen population. Eight populations of North American tetraploids were sampled in addition to seven populations of Eurasian tetraploids and seven populations of Eurasian diploids (Table 2). Each leaf was immediately cut in half and the leaf tip was placed in a 5 mL vial containing RNAlater solution (Ambion, Austin TX). These samples were stored at -20°C for approximately four days, after which they were shipped on dry ice to Colorado State University. Upon arrival samples were placed at -20°C for storage.

### Candidate gene choice

The *C. stoebe *EST library was found to contain a variety of unigenes that share sequence homology with known genes that are involved in plant secondary metabolism and defense response. Many of these unigenes are reported in Broz et al. 2007 [15]. The *C. stoebe *EST library was created from root and shoot tissues of greenhouse-grown plants in rosette form, and represents seven introduced populations [15].

Although multiple candidate unigenes were selected for amplification in an initial analysis, only a small amount of primer sets resulted in reproducible amplification of a single product from *C. stoebe *cDNA (data not shown). Therefore only five candidate genes related to secondary metabolism or defense were quantified in the final analysis (Table 3).

**Table 3:** Primer information table.

**Name (Unigene ID)**	**Primer sequence (5'-3')**	**homologs**	**references**
**Secondary Metabolism**			

PAL 1 phenylalanine ammonia lyase (03772, 01487, 04157, 00435, 00996)	GAAATGGACCCGTTGCAGAAGCC GCTTCGGCTGTTTTTCTTGCGGAAAT	PAL 1 *Arabidopsis* At2g37040 *Lactuca* AAL55242	Olsen et al. 2008 [56] Rookes et al. 2003 [57] Winkel-
PAL 2a (00151)	AGCTCCACCCCTCGAGATTC GTCACCTTCTCACCGGTCAA	PAL 2 *Arabidopsis* At3g53260 *Lactuca *AAO13347	Shirley 2001 [34]
PAL 2b (04127)	ATCGCGAGTACTTCTTCGCC GTCACCTTCTCACCGGTCAA	PAL 2 *Arabidopsis* At3g53260 *Lactuca *AAO13347	La Camera et al. 2004 [32]

**Defense-related**			

Chitinase (00271, 03889, 03038, 04202, 03133)	TGGCTCCATCGTTACTGCATCTG AGTTGTGGGATAGCTGGATAGGTC	Chitinase *Helianthus* AAB57694 Chitinase, class II *Arabidopsis *At4g01700	Kasprzewska 2003 [37] Jwa et al. 2006 [36]
Glucanase (01113, 00896, 00032)	CGACCCGGTTAACATCAAGCTCG CGTCGAAAACTCCGTCGTCTTACC	Beta-1, 3-glucanase *Arabidopsis* At4g14080	Doxey et al. 2007 [35]

**Standards**			

Actin (01058)	ACCAACATGAGAACAACCGATAC TCACACTGGTGTCATGGTCGGAAT	Actin *Gossypium hirsutum* AAP73454	
Cytochrome C oxidase (Weller et al. 2000)	CGTCGCATTCCAGATTATCCA CAACTACGGATATATAAGAGCCAAAACTG		Weller et al. 2000 [60]
Ubiqutin	ACAACATCCAGAAGGAGTCC GCAACACAGCAAGCTTAACC		

The annotation of each *Centaurea stoebe *unigene(s) is given followed by Unigene ID numbers in parentheses (publicly accessible from the PLAN database, , project 30060). For each annotation, forward primer sequence is listed first and reverse primer sequence is listed second. The top BLAST hits (annotation, species, accession number) for each unigene are given in the column "homologs," and references describing information about the genes or gene families are given in the right column.

Three distinct *C. stoebe *unigene homologs encoding phenylalanine ammonia lyase (PAL) were chosen to represent an important subset of secondary metabolism-related genes (PAL1, PAL2a and PAL2b). One set of unigenes had top BLAST hits to PAL1 sequences from *Lactuca sativa *and *Arabidopsis thaliana *(AAL55242 and At2g37040, respectively), and the other two unigenes had top hits to PAL2 sequences from the same organisms (AAO13347 and At3g53260) [56,57], but were distinct from each other upon sequence alignment. In addition, unigenes encoding a class II acidic chitinase (top BLAST hit *Helianthus annuus *chitinase AAB57694) and a beta-1,3-glucanase (top BLAST hit *A. thaliana *endo-glucanase At4g14080) were chosen to represent a subset of defense-related genes (Table 3).

The *C. stoebe *EST library was found to contain six transposable element homologs [15]. Two unigenes encoding transposable elements were initially chosen to analyze the potential for active transposition, which could potentially facilitate rapid evolution. These had top BLAST hits to *Oryza sativa *japonica sequences ABB46630, a CACTA Enhancer Suppressor Mutator (En/Spm) subclass transposon and ABA99201, a mutator subclass transposon (Table 3). Both are type II transposons that move directly as DNA elements through a 'cut and paste' mechanism [58]. Only the CACTA transposon gave reliable Q-PCR results, thus it is the only transposable element listed in the final expression analysis. Transcript accumulation of RAD, involved in homologous recombination and double strand break repair [45], was also analyzed. This sequence was identified by BLAST search and was not derived from the *C. stoebe *EST library. Three housekeeping genes; actin, ubiquitin, and cytochrome c oxidase were also analyzed as controls to normalize the expression of candidate genes (Table 3).

### Gene expression analysis

#### RNA extraction and cDNA synthesis

Approximately 100 mg of each leaf sample (leaf tip) was removed from the RNAlater solution and quickly blotted on filter paper to remove excess liquid. Tissue was immediately frozen in liquid nitrogen and pulverized using a disposable pestle. RNA was isolated using Trizol reagent with its associated protocol (Invitrogen, Carlsbad CA). RNA pellets were resuspended in 30 μL RNase free water, and total RNA was quantified using a NanoDrop spectrophotometer (Wilmington DE). RNA samples were all diluted to the same concentration using RNase free water. RNA was treated with DNase to remove any genomic DNA contamination, and concentrations were re-evaluated using a NanoDrop spectrophotometer (Wilmington DE). Equal amounts of RNA from each sample were then individually translated into cDNA using reverse transcriptase, following a protocol from Invitrogen (Carlsbad CA). Samples were randomized in their preparation, such that RNA from plants from the same population (four plants tested per population) would not all be extracted on the same day.

#### Quantitative PCR

Candidate unigenes were chosen from the *C. stoebe *EST library based on a keyword search using the PLAN database (Table 3, [15,59]). Gene specific primers were designed to amplify a 200–600 basepair region of each candidate *C. stoebe *unigene sequence (Table 3). Initially, specific primer sets were designed for a wide array of genes potentially involved in constitutive defense or secondary metabolism. However, many resulted in either poor amplification or amplification of multiple *C. stoebe *cDNAs, so these were not used in the final Q-PCR analysis. Successful primer sets included those for three distinct transcripts of phenylalanine ammonia lyase (PAL1, PAL2a and PAL2b), a chitinase, a glucanase, a transposable element and a DNA repair enzyme (Table 3). Amplification of each of these transcripts resulted in a single band visualized using agarose gel electrophoresis and each reaction produced a single peak in the Q-PCR melting temperature (Tm) curve, suggestive of a single product. An additional transposable element was successfully amplified in preliminary experiments, but was expressed to a very low extent in the experimental plant samples.

When multiple unigenes had the same annotation, nucleotide sequences were aligned using the DNA alignment program in CLC Free Workbench (Cambridge MA) to determine similarities. Unigenes with over 90% similarity (after removing the terminal 100 bases in case of sequencing error) were grouped together under one annotation, and primers were designed to the alignments. When the ESTs were originally clustered to form unigenes, they had to have an overlap of at least 40 bp and at least 94% sequence identity to be clustered together. The reason some unigenes were grouped in this analysis, but not in the original clustering analysis, is likely due to sequencing errors at the terminal (3') ends of the ESTs, which exhibited the largest amount of variability. In this analysis the terminal 100 bp of sequence was removed, such that only the most reliable sequence information was included. In addition, a few single base changes within similar ESTs were identified and these may represent either sequencing errors or natural polymorphisms. In addition, three potential housekeeping genes were analyzed as controls: actin (*C. stoebe *unigene 01058, top BLAST hit AAP73454, *Gossypium hirsutum*) cytochrome c oxidase (originally designed for *Solanum tuberosum *cv Cara, [60]), and ubiquitin (originally designed for *Nicotiana*). All primer sets amplified a single product from *C. stoebe *cDNA.

All reactions were run and analyzed using the BioRad iCycler software (Hercules CA). A standard curve was created for each primer set using serial dilutions (concentrations of 5–625 ng/μL) of cDNA prepared from leaves of a greenhouse-grown *C. stoebe *plant (fresh tissue was frozen in liquid nitrogen, and RNA extraction and cDNA synthesis followed the protocol above), and negative controls using water instead of template were run for all reactions. The optimal annealing temperature for all primer sets was determined empirically, with all sets working well at an annealing temperature of 55°C. All PCR reactions had a final volume of 20 μL and contained 10 μL of 2× Jumpstart cyber green reaction mix, 0.2 μL 1 μM flourescein, 2.4 μL 25 mM MgCl_2_, 0.2 μL 10 μM forward primer, 0.2 μL 10 μM reverse primer, 2 μL template (20 ng/μL) and 5 μL sterile H_2_O. Reactions conditions for PCR were as follows: 95°C 30 seconds, 55°C 30 seconds, 72°C 30 seconds, for 40 cycles.

For each sample, total RNA (ng/μL) was estimated using the appropriate standard curves and normalized using the geometric mean of actin, cox, and ubiquitin, as suggested in Vandesompele et al. (2002) [61]. Any expression levels that fell below the standard curve for either the gene of interest or the three housekeeping gene standards were removed from the analysis.

### Statistical analyses

In order to account for potential genetic variation within each geo-cytotype (native diploid, native tetraploids, and invasive tetraploid), three to four plants from a number of geographic populations (seven native diploid, seven native tetraploid, and eight invasive tetraploid respectively) were included in this study. We were interested in two *a priori *comparisons for all collected data; native tetraploid versus invasive tetraploid, and native tetraploid versus native dipoid. Differences between geo-cytotypes for gene expression (log cDNA) and for plant characteristics were tested using the MIXED model procedure in SAS (vers 9.1) with geo-cytotype as a fixed variable and population as a random variable. When treating population as a fixed variable, no significant differences between populations within any of the three geo-cytotypes were detected at the p < 0.1 level in any of the analyses. Fisher's LSD was used for pair-wise comparisons of LSmeans to determine significant effects (p < 0.05) for the two pre-planned comparisons. For pair-wise comparisons, the degrees of freedom for all gene expression analysis was equal to 20, and for plant characteristics degrees of freedom are as follows; height = 18, flowering & biomass index = 19, mortality = 17, new rosettes = 15, capitula = 11. Using a Bonferroni multiple comparisons adjustment, the p-values for all reported comparisons remain significant at the p < 0.05 level, except for the chitinase gene expression when comparing native tetraploids to invasive tetraploids (p = 0.089) and the number of new rosettes when comparing native diploids to native tetraploids (p = 0.061). A similar mixed model was run for both gene expression data and plant performance data. All reported values are LSmeans and pooled standard errors.

### Simulation of seed production

Total seed production over time was simulated for *C. stoebe *geo-cytotypes to understand possible differences in fecundity over multiple generations. Data was used from the plant performance analysis for each geo-cytotype (see Additional File 1: Table 1). The simulation followed a cohort of 1000 plants over fifteen generations (years) assuming that the number of flowering plants for each generation was 75.2, 82.1, and 44.3% (invasive tetraploid, native tetraploid and native diploid, respectively) of the total population (Figure 1E); and each generation the number of flowering plants declined according to a mortality rate of 7.3, 23.6, and 62.3% (invasive tetraploid, native tetraploid and native diploid, respectively) as shown in Figure 1F. For each flowering plant, the total number of seeds was estimated as the product of the number of new rosettes per plant (5.88, 5.75, and 2.8 for invasive tetraploid, native tetraploid and native diploid, respectively; Figure 1D), number of capitula per rosette (14.6, 18.6, and 15.7 for invasive tetraploid, native tetraploid and native diploid, respectively; Figure 1C), and 30 seeds per capitula [17]. See Additional File 1: Table 1 for the LSmean values used.

## Authors' contributions

AB designed and carried out tissue sampling, gene choice, gene expression experiment and data analysis, drafted manuscript. DK carried out gene expression experiment and data analysis, edited manuscript. GB designed and carried out greenhouse experiments and data collection, edited manuscript. HMS designed greenhouse experiments, edited manuscript. JV designed gene expression experiment, edited manuscript. All authors read and approved the final manuscript.

## Supplementary Material

Additional file 1 **Measurements of plant performance and life-cycle traits for *C. stoebe *geo-cytotypes, statistical values**. *C. stoebe *plants were grown from seed in a common greenhouse environment. Plants were measured for leaf length and leaf number while in rosette form, and these values were multiplied to obtain an early indicator of biomass. After bolting, stem height of each bolting plant was measured the day the first flower opened and the number of capitula per plant were counted after the stems had senesced. The number of newly formed rosettes after flowering, the percent of flowering individuals, and the percent mortality after flowering were monitored. Legend; 2× EU, native Eurasian diploid populations; 4× EU, native Eurasian tetraploid populations; 4× US, invasive North American tetraploids. Significant differences in plant traits were determined for geo-cytotypes of interest (EU 2× versus EU 4× and EU 4× versus US 4×) using pair-wise comparisons of LSmeans. Reported values are LSmeans. Fisher's LSD and absolute t values are reported for each pair-wise comparison. Click here for file

Additional file 2 **Housekeeping gene expression profiles of *C. stoebe *geo-cytotypes**. Standards used to create normalization factors for analysis of genes of interest. For each sample, total RNA (ng/ul) was estimated using the appropriate standard curve and a normalization factor was calculated based on the geometric mean of all three standards, as suggested in Vandersompele et al. 2002 [61]. Significant differences in gene expression (log cDNA) were determined for geo-cytotypes of interest (EU 2× versus EU 4× and EU 4× versus US 4×) using pair-wise comparisons of LSmeans. Bars represent back-transformed LSmeans and standard errors. Fisher's LSD was used for pair-wise mean comparisons, and values are reported in Table 1. Different letters above the columns indicate significant differences (P < 0.05) between pairs of geo-cytotypes. Legend; 2× EU, native Eurasian diploid populations; 4× EU, native Eurasian tetraploid populations; 4× US, invasive North American tetraploids. Click here for file
