# Supplementary material for: Plant origin and ploidy influence gene expression and life cycle characteristics in an invasive weed
Source: BMC Plant Biol. 2009 Mar 23;9:33. doi: 10.1186/1471-2229-9-33 (PMC2670832; doi:10.1186/1471-2229-9-33)
Supplement: Additional file 1 — Measurements of plant performance and life-cycle traits for C. stoebe geo-cytotypes, statistical values. C. stoebe plants were grown from seed in a common greenhouse environment. Plants were measured for leaf length and leaf number while in rosette form, and these values were multiplied to obtain an early indicator of biomass. After bolting, stem height of each bolting plant was measured the day the first flower opened and the number of capitula per plant were counted after the stems had senesced. The number of newly formed rosettes after flowering, the percent of flowering individuals, and the percent mortality after flowering were monitored. Legend; 2× EU, native Eurasian diploid populations; 4× EU, native Eurasian tetraploid populations; 4× US, invasive North American tetraploids. Significant differences in plant traits were determined for geo-cytotypes of interest (EU 2× versus EU 4× and EU 4× versus US 4×) using pair-wise comparisons of LSmeans. Reported values are LSmeans. Fisher's LSD and absolute t values are reported for each pair-wise comparison. [file 1471-2229-9-33-S1.doc]

**Measurements of plant performance and life-cycle traits for *C. stoebe* geo-cytotypes, statistical values.**

|  | **EU 2x vs EU 4x** | | **Measure of plant performance** | | | **EU 4x vs US 4x** | |
| --- | --- | --- | --- | --- | --- | --- | --- |
| **Trait** | **t** | **p-value** | **EU 2x** | **EU 4x** | **US 4x** | **t** | **p-value** |
| **Biomass index (cm)** | 0.72 | 0.479 | 95.47 ^a^ | 86.07 ^a^ | 72.01 ^a^ | 1.12 | **0.278** |
| **Height (cm)** | 0.96 | 0.348 | 55.43 ^a^ | 55.97 ^a^ | 55.60 ^a^ | 0.63 | **0.538** |
| **Capitula (#/rosette)** | 0.76 | 0.461 | 15.67 ^a^ | 18.57 ^a^ | 14.57 ^a^ | 1.36 | **0.201** |
| **New Rosettes (#/plant)** | **2.39** | **0.036** | **2.80 ^a^** | 5.75 ^b^ | 5.88 ^a^ | 0.74 | **0.857** |
| **Flowering plants (%)** | **2.72** | **0.0137** | **44.34 ^a^** | 82.14 ^b^ | 75.15 ^b^ | 0.52 | **0.608** |
| **Mortality rate (%)** | **2.78** | **0.0127** | **62.31 ^a^** | **23.55 ^b^** | **7.34 ^b^** | **1.44** | **0.1685** |

*C. stoebe* plants were grown from seed in a common greenhouse environment. Plants were measured for leaf length and leaf number while in rosette form, and these values were multiplied to obtain an early indicator of biomass. After bolting, stem height (B) of each bolting plant was measured the day the first flower opened and the number of capitula per plant (C) were counted after the stems had senesced. The number of newly formed rosettes after flowering (D), the percent of flowering individuals (E), and the percent mortality after flowering (F) were monitored. Legend; 2X EU, native Eurasian diploid populations; 4X EU, native Eurasian tetraploid populations; 4X US, invasive North American tetraploids. Significant differences in plant traits were determined for geo-cytotypes of interest (EU 2x versus EU 4x and EU 4x versus US 4x) using pair-wise comparisons of LSmeans. Reported values are LSmeans. Fisher’s LSD and absolute t values are reported for each pair-wise comparison.
