# Supplementary material for: Plant origin and ploidy influence gene expression and life cycle characteristics in an invasive weed
Source: BMC Plant Biol. 2009 Mar 23;9:33. doi: 10.1186/1471-2229-9-33 (PMC2670832; doi:10.1186/1471-2229-9-33)
Supplement: Supplementary file 2 [file 1471-2229-9-33-S2.doc]

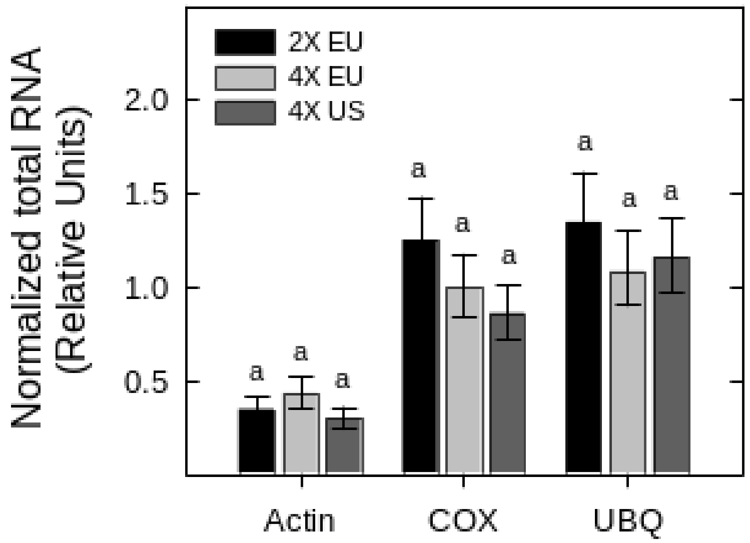

Normalized total RNA
(Relative Units)
2X EU
4X EU
4X US
2.0
1.5
1.0
0.5
a
Actin
COX
UBQ
